# Role of Milk-Derived Antibacterial Peptides in Modern Food Biotechnology: Their Synthesis, Applications and Future Perspectives

**DOI:** 10.3390/biom8040110

**Published:** 2018-10-05

**Authors:** Muhammad Usman Khan, Maryam Pirzadeh, Carola Yvette Förster, Sergey Shityakov, Mohammad Ali Shariati

**Affiliations:** 1 Bioproducts Sciences and Engineering Laboratory (BSEL), Washington State University, Richland, WA 99354, USA; muhammadusman.khan@wsu.edu; 2 Department of Energy Systems Engineering, Faculty of Agricultural Engineering and Technology, University of Agriculture, Faisalabad 38000, Pakistan; 3 Department of Food Science and Technology, Faculty of Agriculture, Sarvestan Branch, Islamic Azad University, Sarvestan 73451-173, Iran; pirzadeh224@yahoo.com; 4 Department of Anesthesia and Critical Care, University of Würzburg, 97080 Würzburg, Germany; Foerster_C@ukw.de; 5 Laboratory of Biocontrol and Antimicrobial Resistance, Orel State University Named After I.S. Turgenev, 302026 Orel, Russia

**Keywords:** milk proteins, bioactive peptide, antibacterial activity, fermentation, protein hydrolysis, recombinant DNA, peptide synthesis

## Abstract

Milk-derived antibacterial peptides (ABPs) are protein fragments with a positive influence on the functions and conditions of a living organism. Milk-derived ABPs have several useful properties important for human health, comprising a significant antibacterial effect against various pathogens, but contain toxic side-effects. These compounds are mainly produced from milk proteins via fermentation and protein hydrolysis. However, they can also be produced using recombinant DNA techniques or organic synthesis. This review describes the role of milk-derived ABPs in modern food biotechnology with an emphasis on their synthesis and applications. Additionally, we also discuss the mechanisms of action and the main bioproperties of ABPs. Finally, we explore future perspectives for improving ABP physicochemical properties and diminishing their toxic side-effects.

## 1. Introduction

Milk-derived antibacterial peptides (ABPs) are a plentiful group of biochemical substances produced from milk with a molecular weight below 10 kD [1,2,3,4]. Most of these ABP compounds are produced by organic synthesis, in vitro via enzymatic proteolysis (fermentation or protein hydrolysis) of milk proteins, in vivo by molecular cloning using natural sequences [5].

Milk of all mammalian species as a heterogeneous mixture is produced by lacteal glands [6,7]. It comprises approximately 3.5% of total protein fraction, including 80% of casein and the rest of the whey proteins, which exhibits a variety of biochemical and physiological properties [8,9,10]. In turn, casein and whey fractions have been subdivided into α-, β- and κ-caseins, and whey lactalbumins and lactoglobulins with some additional proteins, such as immunoglobulins, enzymes, and mineral-binding proteins [11,12,13].

The various multifunctional properties of milk-derived ABPs have been extensively studied to investigate their positive impact on human health [14,15]. Primarily, this naturally occurring bioactive peptides are low-density molecules (5–90 amino acids) representing their bioactivity features only if they are separated from the parental proteins [16] being produced in several different forms [17]. 

All ABPs in this review are mainly divided into four classified groups: (i) milk-derived, such as isracidin αs1 f(1–23) and lactoferricin f(17–41); (ii) whey-derived peptides such as β-lactoglobulin f(15–20); (iii) casein-derived, such as κ-casecidin and its partial peptide fragments, and (iv) lysozyme-derived ABPs.

The degree of ABP antibacterial activities depends on the biophysical features such as negatively and positively charged groups of peptides, molecular size, conformational and hydrophobic properties [18]. Additionally, some milk-derived ABPs may exploit routine regulating activities in the human body as products of proteolytic reactions, such as enzymatic hydrolysis and fermentations [19]. Proteolytic enzymes from the dairy products, such as milk plasmin might hydrolyze proteins to release ABPs during milk processing and storage [20]. Moreover, many types of bacteria, which reside in the gastrointestinal tract of animals and humans, can produce bioactive ABPs from milk during its digestion [21].

In general, milk-derived ABPs have drawn much attention of the scientific community worldwide due to their biological versatility with the ability to formulate them with pharmaceutical ingredients and health-promoting food supplements [22]. Moreover, these peptides are also prone to polymorphism, so they occur in multiple isoforms [23,24,25,26]. Here, we discuss the role of milk-derived ABPs in modern food biotechnology, focusing on their application, production, and future perspectives.

## 2. Mechanism of Action 

Antibacterial activity of ABPs depends on their cationic and hydrophobic amino acid composition [27] with a mild positive charge (+4) under physiological conditions (Table 1). Most of the charged ABPs disrupt lipid membranes altering their permeability and transport properties [27,28,29]. In particular, positively charged amino acids are extremely active against Gram-positive and Gram-negative bacteria [30,31,32,33,34,35,36,37,38,39,40]. In comparison to conventional antibiotics, ABPs have considered interacting with bacterial DNA and RNA [32,41], forming a hydrogen bond with substances such as 3,4-dihydroxyphenylalanine [33,42] or sodium chloride [30,39]. This ABPs antibacterial action would lead to the membrane dissolution or a specific binding to nucleic acids [34,35,43,44].

## 3. Milk-Derived Antibacterial Peptides 

A diversity of peptides comes from the different food protein sources (so-called functional food) with some specific properties such as antibacterial, anti-carcinogenic, hormone-tropic, immunomodulatory and antihypertensive effects [36,37,38,39]. The main source of bioactive peptides is dairy products, such as milk [40,41,42,43].

Milk contains different nutrient as an entire source of various proteins and peptides [44,45,46]. Since milk contains a wide spectrum of peptides, the concept of consideration of milk as a high nutritive source increased its attractions among consumers. The lower size of peptides provides them with the ability of quick diffusion into the cell membrane of pathogens and makes the leaky as direct suppressing and antibacterial actions [47,48,49,50]. Overall, naturally occurring milk peptides might even diminish the time span of disease, which is the result of their antibacterial effect on pathogens [51,52].

Milk is a well-balanced protein source, containing two main fractions, such as casein and whey [53] to satisfy mammalian offspring necessitates. Milk has also immunological peptides as bacterial inhibitors to decrease the growth of pathogens. Furthermore, proteins such as lactoferrin (Lf), lactoperoxidase, and lysozyme are among those protecting ingredients [54,55].

Considering milk as a primary nutritional source presenting of more than 10,000 nutritional compounds in milk has given a perspective of being recognized as a functional food. Beyond basic functional compounds, bioactive peptides are the main group of milk health affecting constituents, which provides an array of functional activities with treating properties such as reducing grade inflammation, antibacterial effects, etc. [56,57,58].

Amino acids and nitrogen constituents are the two main targets of milk consumption [59]. Milk proteins are precipitated in its isoelectric pH 4.6 at 20 °C, following by the fragmentation mainly to β-lactoglobulin-β-LG-(7–12% of total skim milk protein), -lactalbumin-LA-(2–5% of skim milk total protein), serum albumin (SA), Immunoglobulins-Ig, lactotransferrin (lactoferrin-Lf) and β2-microglobulin. Table 2 and Table 3 summarize some ABPs with the corresponding minimal inhibitory concentrations together with peptide production approaches and antibacterial effects.

## 4. Whey-Derived Antibacterial Peptides 

Lactoferrin, is a glycoprotein, which consists of small fractions of milk proteins. This protein produces structural fragments proteolytic treatment than create antibacterial potencies as ABPs against various bacterial pathogens [50,71,72]. The antibacterial properties of Lf are dependent on its charge, hydrophobic properties, or secondary structure as it has is a higher affinity for iron atom than most of the proteins in *Streptococcus mutans*, *Vibrio cholerae*, *Escherichia coli*, and *Legionella pneumophila*. In fact, Lf follows multiple mechanisms in suppressing pathogens [64,73]. Some of them are based on positively charged arginine and tryptophan residues that facilitate the interaction with negative charges of lipopolysaccharides of the lipid membrane, leading to a bacterial death.

Lactoferrin, itself has pathogenic properties, acting as a chelating agent by capturing the ions (apo-Lf) important for bacteria [31,74,75].

Lactoferrin was identified in two protein variants as human (Lf H) and bovine (Lf B), with antibacterial potentials on both positive and negative bacteria strains. The N1-domain of Lf acts against pathogens such as *Bacillus subtilis*, *Escherichia coli*, and *Pseudomonas aeruginosa*, but effective against the fermentation bacteria [76]. Unlike bovine whey, in which β-lactoglobulin contains 50% of whole protein, it has no presence in human milk. Trypsin digestion method of β-lactoglobulin produces four peptide fragments (f), including f(15–20), f(25–40), f(78–83) and f(92–100) active mainly against gram-positive bacteria. On the other hand, α-lactalbumin produces anti-gram-positive peptides after trypsin or chymotrypsin digestion [76].

## 5. Casein-Derived Antibacterial Peptides 

Casein, comprising 80% of milk protein, is believed to be the main protein fraction of milk [77]. Although casein itself exhibits no any antibacterial effect, the ABPs, which released from its enzymatic digestion, may exert antibacterial activity as functional oligopeptides [78]. The digesting method of casein by chymosin at neutral pH produces some antibacterial agents, such as casecidin, lactenin, and isracidin, inhibiting the growth of some strains in vitro [79] by the N-terminal fragment of αs1-casein [79]. Additionally, chymosin proteolytic digestion may also generate other fragments, comprising caseicin A and B that inhibit several pathogens, among them Staphylococcus aureus, *Sarcina lutea*, *Bacillus subtilis*, *Diplococcus pneumoniae*, and *Streptococcus pyogenes* [73,80]. These two fragments from αs1-casein with the ability to inhibit *Cronobacter sakazakii*, *Salmonella* and *Klebsiella*, and Gram-positive *Staphylococcus aureus* in the powdered food [81,82,83,84]. Furthermore, casecidin-I as a cationic αs2-casein-derived peptide (165–203 amino acids) could suppress the growth of Gram-negative (*E. coli*) and Gram-positive (*Staphylococcus carnosus*) bacteria [85]. Some other αS2-casein-derived peptides f(181–207), f(175–207), f(164–207) possess antibacterial properties versus pathogenic bacteria [65,73].

On the other hand, kappacin (nonglycosylated κ-casein), is a peptide of human milk acidification, belonging to the phosphorylated form of ABP. Kappacin shows bactericidal potential against Gram-positive (*Streptococcus mutans*) and Gram-negative (*Porphyromonas gingivals*) bacteria, whereas its non-phosphorylated and glycosylated forms might have no effect on some groups of bacteria such as Streptococcus mutans [86]. κ-casecidin and its partial peptide fragments are produced via trypsin digestion method of casein to reduce the growth of *S. aureus*, *E. coli* and *S. typhimurium*. Casein macropeptide (CMP) derived from κ-casecidin can disrupt replication of invasive bacteria through the specific binding to their receptors on the cell wall [85]. In addition, CMP also prevents the propagation of microflora, forming dental plaques caused by *Streptococcus mutans* [86].

## 6. Lysozyme-Derived Antibacterial Peptides 

There are different types of lysozyme available in milk whose properties differ from each other, depending on their structure, physiochemical properties, and the ability of binding to calcium [87]. In fact, milk-derived lysozyme has a significant potential of being recognized as an antibacterial compound through its lysis reaction. Some lysozyme-derived ABPs, such as RAWVAWR-NH_2_ and IVSDGNGMNAWVAWR-NH_2_, were found to exhibit antibacterial activity with the ability to rapidly enter both *E. coli* and *Staphylococcus aureus* [88]. These peptides can cause a significant perturbation of membranes due to their strong affinity to the lipids with negatively charged head groups [88]. Additionally, 87–114 and 87–115 amino acid residues of chicken and human lysozyme were tested for bactericidal activity against Gram-positive and Gram-negative bacteria in the attempt to design novel ABPs [89]. However, ion concentration changes of sodium, potassium, ammonium, magnesium, and calcium might influence the lysozyme and probably its ABPs antibacterial activity by a reduction of their minimum inhibitory concentration (MIC) in the experiment [87].

## 7. Production of Antibacterial Peptides 

In general, most ABPs belong to biologically active protein fragments that are mainly being produced with some modifications either by enzymatic (proteinases and peptidase) digestion and fermentation processes, or by lactic acid bacteria (LAB) hydrolysis, not excluding the application of some peptidases from other organisms, such as animals, plants or even fungi, etc. [90]. In this regard, four main strategies were elaborated and optimized for the industrial production of ABPs: fermentation, protein hydrolysis with extracellular enzymes, recombinant DNA method, and organic synthesis (Table 4).

### 7.1. Fermentation by Lactic Acid Bacteria

Fermentation is a process in which some peptidases are produced by LABs decomposing proteins into their structural fragments. Currently, this methodology is considered outdated for efficient production of functional food [92,93]. Conversely, the LAB usage to produce bioactive peptides such as ABPs from milk proteins is a straightforward process strategy believed to be GRAS, “generally recognized as safe” [93].

Fermentation is used to decompose milk ingredients, producing better taste, smell and color (organoleptic properties) together with bioactive peptides active against *Salmonella enteridi* and *Escherichia coli* [94]. Lysozyme, H_2_O_2_, lactoferrin and various ABPs are known as substances, which reduce blood pressure and stimulate the innate immune system [95]. Moreover, they also act as food preservatives against *Escherichia coli*, *Listeria monocytogenes*, *Staphylococcus aureus*, and *Salmonella typhimurium* [93].

On the other hand, ABPs, as bacteriocins (colicins) may refer to antibacterial peptides mainly from the Gram-positive bacteria (LABs), which secrete these components to the surrounded media and suppress pathogen growth [96,97,98,99]. Therefore, the development of a system introducing sufficient production, distribution, and delivery of ABPs is of primary concern for modern food biotechnology to improve the antibacterial activity of naturally manufactured compounds via bacteria [100,101].

In the fermentation process, LABs can produce lantibiotic bacteriocins, among them nisin, helveticin, lactacin, etc., which are secreted (nisin) by some genera including *Lactococcus lactis*, *Pediococcus acidilactici*, and *P. pentosaceus*. Pediocin is another bacteriocin, which inactivates *L. monocytogenes*, *Enterococcus faecalis*, *S. aureus*, and *C. perfringens*. Furthermore, nisin as a small cationic polypeptide is approved by FAO/WHO to be safe as a food supplement [102,103,104,105,106]. This ABP might prevent pathogenic effects of both types (Gram-positive and negative) of bacteria [20,106].

As protein-based synthetic components, bacteriocin are involved in the suppression of different the Gram-positive and negative bacteria via the interaction with lipid membranes due to their amphiphilic and hydrophobic properties [107]. Natamycin, another bacteriocin, a polyene antifungal agent produced by *Streptomyces natalensis*, is effective against molds and yeasts, but it has mild or no effect on bacteria or viruses [108]. Natamycin has a very low aqueous solubility, therefore, it needs to be applied at high concentration and is effective at very low levels [109]. In particular, reuterin and reutericyclin made by *Lactobacillus reuteri* are highly active against *L. monocytogenes*, *E. coli* (O157:H7), *S. choleraesuis*, *Yersinia enterocolitica*, *Aeromonas hydrophila*, and *Campylobacter jejuni*. Therefore, the development of a system introducing sufficient production, distribution, and delivery of ABPs is of primary concern for modern food biotechnology to improve the antibacterial activity of naturally manufactured compounds via bacteria [100,101].

### 7.2. Protein Hydrolysis with Extracellular Enzymes (Proteases)

Other strategies have been employed in modern food biotechnology to produce more effective ABPs [91]. In particular, the chymosin (rennin) proteolytic reactions of casein have resulted in the formation of some antibacterial peptides, such as isracidin, matching the N-terminal part of αs1-casein [64]. This casein-derived substance remains active against *Staphylococcus aureus* and *Candida albicans* [110]. Similarly, another peptide casiocidine is formed from the αs2-casein protein together with (183–207) and (164–179) fragments, via pepsin proteolytic processing [66,73]. Additionally, κ-casein has two antibacterial fragments after pepsin digestion as the (138–158) and (64–117) fragments named kappacine, which could kill cariogenic bacteria [76]. A proteolytic hydrolysis of β-casein by *Lactobacillus helveticus* PR4 creates the (184–210) fragment with antibacterial properties and the (138–158) fragment active against *Str. mutans*, *E. coli* and *Porphyromonas gingivalis* [76].

Apart from the above-mentioned peptides, other fragments can be formed due to ionic conditions and pH changes, leading to their precipitation without a separation phase [48]. For instance, caseinomacropeptide characterized by the reversed-phase high-performance liquid chromatography coupled with mass spectrometry (HPLC-MS) has antibacterial properties against *Streptococcus mutans*, *Porphyromonas gingivalis* and *Escherichia coli* [68].

Additionally, kappacin as the κ-casein-derived product active against *S. mutans*, *E. coli*, and *Porphyromonas gingivalis* [68] together with chymosin, which was found during proteolytic activities of sodium caseinate [62,111].

Pepsin proteolytic activities have been commonly employed to denature milk proteins and to produce various fragments for further evaluation with HPLC-MS of these oligopeptides, which turned out be active against *Bacillus cereus*, *Staphylococcus aureus*, *Enterococcus faecalis*, and *Escherichia coli* [77].

Two classes of antibacterial peptides are defined of fungal- and bacterial-origin, characterized by the cyclic or branched composition of ABPs [112]. Another classification subdivides them into (i) cryptic peptides as a product of enzymatic reactions, (ii) lantibiotics, such as nisin, (iii) defensin and cathelicidins related to the immune system [113,114]. Most ABPs are derived from native proteins by enzymatic hydrolysis to generate desired peptides or fragments by screening, fractionation, and purification [115].

Sometimes de novo peptide sequencing is needed by mass spectrometry to obtain the predicted ABP structure [116]. However, some peptides in the active fraction are sometimes not biologically active requiring additional bioinformatics analysis to screen them for their biological and physiological activity [117]. Therefore, the ABP purification method implies the use of the ammonium sulfate, which precipitates protein fragments with 80–100% fractional activity [117]. For a supernatant concentration, there are several approaches such as ammonium sulfate concentration adjustment, absorption-desorption technique, and organic solvent extraction [118].

By applying a salting out technique, one could extract bacteriocins of different microorganisms such as LABs [119], *Pediococcus* spp. [120], *Lactococcus* spp. [121], and *Leuconostoc* spp. [122]. In the previous study, the researchers found that the membrane benzylation followed by the dialysis with a cutoff of 2–3.5 kDa resulted in the highest extraction of smaller size bacterocins [118].

Milk-derived peptides from the pepsin digestion are active against a wide range of pathogens [62]. Caseinate fermentation by *L. acidophilus* DPC6026 produces caseicins A, B, and C [81]. Fermentation is one of the cheapest methods for the efficient production of ABPs in comparison to the proteinase approach [91].

Rana and co-authors have already used this method to evaluate and characterized ABP-like peptides from milk fermentation products by *L. rhamnosus* C6 [123]. Additionally, pepsin digestion method might be also useful to ABPs [124], which was confirmed by reversed-phase chromatography and sensitive radial diffusion method to characterize the antibacterial activity of separated fragments present in human milk [124].

Due to the interference in the ABPs identification, a technology named matrix-assisted laser desorption ionization–mass spectrometry (MALDI–MS) can be applied [125]. The technique was successfully implemented to observe a bacteriostatic effect of the human k-casein fragment (63–117) [85]. Additionally, hydrochloric acid can be supplemented as an activation factor for pepsin, trypsin, and chymotrypsin to denature casein with a subsequent release of various ABPs [45,54,126].

### 7.3. Antibacterial Peptides Synthesis by Recombinant DNA Method

Recombinant DNA technology has been widely used as an alternative to the aforementioned techniques to produce ABPs in high amounts [127,128]. This procedure is particularly useful for the synthesis of large ABPs (>150 amino acids) and proteins [129,130,131,132]. The overall strategy relies upon the construction of the ABP coding region with its subsequent cloning into a prokaryotic expression vector, allowing the production of ABP or several peptides, simultaneously. To achieve this goal, *E. coli* cells—the most widely used host—might be implemented as the expression system [133].

Since most ABPs represent a strong antibacterial activity against the expression vector cells and relative sensitivity to proteolytic enzymes, these peptides are usually expressed as fusion proteins to neutralize their inherent toxic properties and improve their expression levels [133]. Compared with isolation from natural sources and organic synthesis methods, the recombinant DNA approach provides the most cost-effective alternative for industrial (large-scale) ABP production. Table 5 summarizes the synthesis of milk-derived ABPs by using recombinant DNA technology.

## 8. Summary and Future Perspectives

In this review, we discuss the role of milk-derived ABPs in modern food biotechnology, focusing on their application and production. Although different methods (fermentation, protein hydrolysis, recombinant DNA technology, and organic synthesis) have been successfully applied to produce various ABPs from milk, their stability and solubility should to be considered. To enhance these features, some formulated excipients, such as amphiphilic cyclodextrins, might be used.

**Figure 1:**
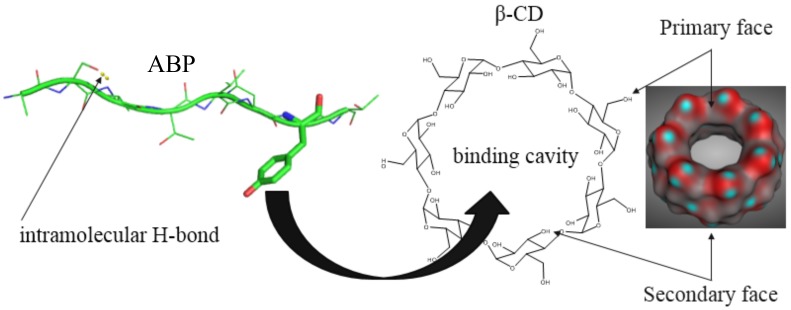
Hypothetical ABP/β-CD (anti-bacterial peptide/β-cyclodextrin) complexation/formulation process via the molecular docking of peptide lipophilic side chains into the hydrophobic β-CD binding cavity.

Cyclodextrins (CDs) are starch by-products of converting enzymes that are composed of a (1, 4)-linked glucopyranose and defined as α, β, γ according to the number of the glucose units (6, 7, and 8) in the molecule [138]. These amphiphilic molecules possess a lipophilic binding cavity that could mediate complexation with ABPs (Figure 1).

In some cases, the antibacterial activity of ABPs might be inhibited when these peptides are exposed to cholesterol [139]. Therefore, any addition of cyclodextrins may diminish this effect due to the cholesterol absorption by CDs [139].

Conversely, CDs are well known for increasing drug-like molecule solubility upon their complexation with the former molecules [140,141]. For instance, in the study of colicin, the β-CD (β-cyclodextrin) addition to the oleic acid (OA) solution provided better OA delivery and insertion into the lipid membrane [142].

The interaction of CDs with the cellular membranes causes its structural change, allowing ABPs to enter the cell [143]. The presence of hydroxyl groups and carbon core in the CD structure divides the molecule into a hydrophilic exterior and a hydrophobic interior as binding cavity [138,144]. Hydrophobic amino acids (mainly tyrosine and tryptophan) with aromatic rings are the driving force of interaction between amphiphilic cyclodextrins and ABPs due to the steric effects (Figure 1).

Another important approach alleviate ABP toxicity can be found in the replacement of highly toxic dimethylformamide and methylpyrrolidone organic solvents via hydrophilic cyclodextrin complexation/formulation of lipophilic peptides or using less toxic solvent analogs, such as 3-methoxy-3-methyl-1-butanol (MMB), PEG-400, glycerol and propylene carbonate.

**Figure 2:**
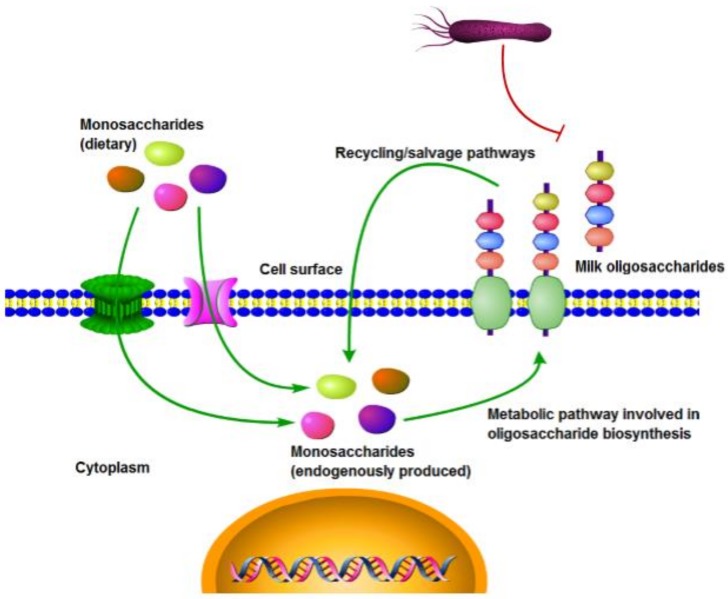
Hypothetical model indicating that dietary monosaccharides (DMs) might be taken up by the intestinal cell and used for the synthesis of cell surface glycoconjugates [145] with modifications). These glycoconjugates and milk oligosaccharides (MOs) might inhibit the adhesion to the cell of bacterial pathogens.

The other strategies might be employed to increase the ABPs efficiency against pathogenic bacteria is to use them in combination with other antibacterials and prebiotics, such as milk oligosaccharides (MOs) [145]. It is well known that some MOs may attenuate pathogens because of their stimulation of the lactic acid and bifidobacteria growth in the gut [146]. Moreover, MOs may defend the human body against pathogens via the creation of entrapment system to inhibit their binding to epithelial cells [145]. This could be achieved by the hypothetical synergistic effect of dietary monosaccharides (DMs) and MOs where DMs might be taken up by the intestinal cell and used for the synthesis of modified cell surface glycoconjugates [145]. These surface glycoconjugates together with MOs might interfere with the adhesion of bacterial pathogens to the cell wall by the inhibition of this process (Figure 2). 

Additional research is needed to investigate the pharmacokinetic/pharmacodynamic parameters of complexed ABPs and their ability to permeate different biological barriers, such as the blood-brain barrier (logBB determination) for more effective treatment of infectious diseases. Finally, the advent of nanobiotechnology allows for the design of highly effective hybrid nanomaterials with synergistic effects of ABPs and nanoparticles, such as metals and their oxides, metal-organic frameworks, and nanoclays, to enhance the biodistributional and barrier properties of ABP formulations.

## Figures and Tables

**Table 1:** Functional activity of milk-derived antibacterial peptides (ABPs) (adopted from [36,45]).

**ABPs**	**Circulatory System**	**Nervous System**	**Immune System**	**Gastrointestinal Tract**	**Functional Peptide**
	Antihypertensive peptides	Opioid peptides	Immunomodulation peptides	Regulatory and enzyme inhibitors	Sensory peptides
	Antithrombotic peptides		Antibacterial peptides	Celiac toxicity	Antioxidative peptides
				Microelement-binding peptides	Surface active peptides

**Table 2:** Minimum inhibitory concentration (MIC) for different fragments of milk-derived ABPs (adopted from [60,61,62,63]).

ABP	MIC	Pathogen
αs2-casein f(151–181)	15.6 μg/mL	*Bacillus subtilis* ATCC6051,
	16.2 μM (62.5 μg/mL)	*Escherichia coli* NEB5α and *E. coli*, ATCC25922
αs2-casein f(182–207)	2.7 μM (8.6 μg/mL)	*B. subtilis* ATCC6051,
	21.4 μM (68.8 μg/mL)	*E. coli* NEB5α,
Lactoferrin	125 mg/mL	*E. coli*,
	250 mg/mL	*Salmonella typhimurium*,
	125 mg/mL	*Salmonella enteritidis*,
	500 mg/mL	*Citrobacter freundii*,
	2.5 mg/mL	*Candida albicans*

**Table 3:** Summary of milk-derived ABPs and their antibacterial effect (adopted from [60]).

ABP	Production	Inhibition	References
Isracidin αs1 f(1–23)	Chymosin digestion	Several microorganisms in vivo and in vitro	[64]
Lactoferrin B f(18–36) and f(17–41/42)	Enzymatic digestion (pepsin and chymosin)	Some Gram (+) and Gram (−) bacteria	[65,66]
Lactoferricin f(17–41)	Enzymatic digestion (pepsin and chymosin)	Some Gram (+) and Gram (−) bacteria, viruses, fungi, and parasites	[65,67]
Lf f(268–284)	Enzymatic digestion (pepsin and chymosin)	*B. subtilis, E. coli, P. aeruginosa*	[68]
αs2 casein f(183–207)	Digestion with pepsin	Some Gram (+) and Gram (−) bacteria	[66]
κ-casein f(106–169) (kappacin)	Digestion with chymosin	*S. mutans*, *E. coli*	[69]
κ-casein f(18–24) and f(30–32) and f(139–146)	Digestion with pepsin	Some Gram (+) and Gram (−) bacteria	[70]
Lf f(1–48) and f(1–47)	Digestion with pepsin	*M. flavus*	[70]
α–La f(1–5) and f(17–31) and f(61–68)	Digestion with chymotrypsin	Some Gram (+) Gram (−) bacteria	[71]
B–Lg f(15–20), f(25–40), f(78–83) and f(92–100)	Digestion with trypsin	Some Gram (+) and Gram (−) bacteria	[71]

**Table 4:** Industrial production of ABPs [91].

Production Method	Production	Scale
Fermentation	Not precise	Laboratory and industrial
Protein hydrolysis	Not precise	Limited to laboratory
Recombinant DNA	Large ABPs (>150 amino acids)	Laboratory and industrial
Organic synthesis	Medium-size ABPs	Laboratory and industrial

**Table 5:** Synthesis of milk-derived ABPs by recombinant DNA technology.

Derivative Antibacterial Peptides	Parental Compound	Expression System	Inhibited Growth	Reference
Lactoferricin B-W10 (LfcinB-W10),	Lactoferricin Lf-(f17–41)	*E. coli* BL21 (DE3).	*S. aureus* ATCC25923	[134]
Lfcin B15-W4,10	Lactoferricin Lf-(f17–31)	*E. coli* BL21 (DE3).	*S. aureus* ATTC25923	[135]
LFT33	Bovine lactoferricin and thanatin (an inducible insect antibacterial peptide)	*E. coli* BL21	Significant antibacterial activity compared to parental compound	[136]
Lactophoricin	Residues 113–135 of proteose-peptone (component 3)	*E. coli* C41 (DE3)	Not mentioned	[137]
